# 1q21.1 microduplication: large verbal–nonverbal performance discrepancy and ddPCR assays of HYDIN/HYDIN2 copy number

**DOI:** 10.1038/s41525-018-0059-2

**Published:** 2018-08-22

**Authors:** Jean Xavier, Bo Zhou, Frédéric Bilan, Xianglong Zhang, Brigitte Gilbert-Dussardier, Sylvie Viaux-Savelon, Reenal Pattni, Steve S. Ho, David Cohen, Douglas F. Levinson, Alexander E. Urban, Claudine Laurent-Levinson

**Affiliations:** 1 Sorbonne Université, Faculté de Médecine, Groupe de Recherche Clinique n°15 (PSYDEV), AP-HP, Hôpital Pitié-Salpêtrière, F-75013 Paris, France; 2 Sorbonne Université, CNRS, Institut des Systèmes Intelligents et Robotiques (ISIR), UMR 7222, F-75252 Paris, France; 3 Department of Psychiatry and Behavioral Sciences, Stanford University, Stanford, CA USA; 4 Service de Génétique, CHU de Poitiers, Poitiers, France; 5 EA3808, Université de Poitiers, Poitiers, France; 6 Centre de Référence des Maladies Rares à expression psychiatrique, Department of Child and Adolescent Psychiatry, AP-HP, Hôpital Pitié-Salpêtrière, Paris, France

## Abstract

Microduplication of chromosome 1q21.1 is observed in ~0.03% of adults. It has a highly variable, incompletely penetrant phenotype that can include intellectual disability, global developmental delay, specific learning disabilities, autism, schizophrenia, heart anomalies and dysmorphic features. We evaluated a 10-year-old-male with a 1q21.1 duplication by CGH microarray. He presented with major attention deficits, phonological dysphasia, poor fine motor skills, dysmorphia and mild autistic features, but not the typical macrocephaly. Neuropsychiatric evaluation demonstrated a novel phenotype: an unusually large discrepancy between non-verbal capacities (borderline-impaired WISC-IV index scores of 70 for Working Memory and 68 for Processing Speed) vs. strong verbal skills – scores of 126 for Verbal Comprehension (superior) and 111 for Perceptual Reasoning (normal). *HYDIN2* has been hypothesized to underlie macrocephaly and perhaps cognitive deficits in this syndrome, but assessment of *HYDIN2* copy number by microarray is difficult because of extensive segmental duplications. We performed whole-genome sequencing which supported *HYDIN2* duplication (chr1:146,370,001-148,590,000, 2.22 Mb, hg38). To evaluate copy number more rigorously we developed droplet digital PCR assays of *HYDIN2* (targeting unique 1 kb and 6 kb insertions) and its paralog *HYDIN* (targeting a unique 154 bp segment outside the *HYDIN2* overlap). In an independent cohort, ddPCR was concordant with previous microarray data. Duplication of *HYDIN2* was confirmed in the patient by ddPCR. This case demonstrates that a large discrepancy of verbal and non-verbal abilities can occur in 1q21.1 duplication syndrome, but it remains unclear whether this has a specific genomic basis. These ddPCR assays may be useful for future research on *HYDIN2* copy number.

## Introduction

Microduplication of 1q21.1 is observed in ~0.03% of adults.^1,2^ It has a variable and incompletely penetrant phenotype including diverse dysmorphic features, macrocephaly, congenital heart disease (tetralogy of Fallot), connective tissue abnormalities and neuropsychiatric disorders including learning disabilities, developmental delay, autism spectrum disorders (ASD), attention deficit hyperactivity disorder (ADHD) and schizophrenia.^2–8^

We describe here a 10-year-old boy with a 1q21.1 microduplication with a predominantly neuropsychiatric and cognitive phenotype. In contrast with the low IQ that is frequently observed, he had a previously-unreported feature: a striking discrepancy between verbal (superior range) and non-verbal (borderline) cognitive performance.

Following a comprehensive medical and neuropsychiatric evaluation, we carried out whole-genome sequencing (WGS) to search for a genomic basis for the phenotype. We also developed droplet digital PCR (ddPCR) assays for copy number (CN) of *HYDIN2* (1q21.1) and its 16q22.2 paralog *HYDIN*, because *HYDIN2* was previously hypothesized to play a role in changes in brain size (normal in this case) and other neuropsychiatric features.^5^ *HYDIN2* is classified as a pseudogene but is expressed in CNS.^5,9^ Most 1q21.1 CNVs form between segmental duplication (SD) regions BP2-BP3 (thrombocytopenia-absent radius syndrome; TAR) or the larger, more distal BP3-BP4 region (producing neuropsychiatric and other features).^3,10^ Typical distal boundaries are ~144.6–146.3 Mb (hg18)^4,7^ (hg19: 145.8–147.8).

## Case report

T is a boy with two siblings who were in good health. The primary guardian provided written informed consent. There is no known parental consanguinity, and no known maternal family history of psychiatric disorder or learning disability. Father was unavailable. T was born by vaginal delivery at 38 weeks + 5 days of amenorrhea. Pregnancy was uncomplicated, without neonatal resuscitation or jaundice. Mother was 23 and father 27 at conception. Birth weight was 3480 g, length 49 cm and head circumference 35 cm (all normal). Apgar scores were 10/10 (1/5 min). At age 10, height was 138 cM (50^th^ percentile), weight was 30 kg (below the 25^th^ percentile) and head circumference was 54 cm (+0.5 SD). He was examined by a medical geneticist at age 5½ for language delay. A comprehensive neuropsychiatric evaluation for behavioral problems was conducted beginning at 9 years-11 months.

### Developmental history

He walked at 24 months and had difficulty with fine motor skills (handwriting, drawing), coordination (ongoing difficulty tying shoes, buttoning clothing, riding a bicycle), speech and language delay (first words ~18 months; combinations of words ~3 years) and articulation (e.g., “totolat” instead of “chocolat”). He was toilet trained by age 6. He began speech and occupational therapy at age 4. At 10 he was attending a therapeutic school for learning disabilities. The school described him as a non-reader with articulation problems but with sustained oral expression. He had trouble tracking time, was anxious and impulsive with poor attention and memory, and was socially isolated.

### Medical history

As an infant he had repeated bronchitis and ear infections, treated with tympanostomy tubes. At age 7½ he developed difficulty falling asleep and repeated awakenings every 2–3 h. Physical examinations of the heart, lungs, abdomen and eyes were repeatedly normal. No genito-urinary abnormalities were recorded (no structural evaluations were completed).

### Morphological features

At ages 5½ and again at 10 he was described as having a long trunk, a long, square face, widely spaced eyes (hypertelorism) and large cheeks. Philtrum was long and well defined, upper lip was thin, with a flattened nose and without the normal protrusion of the middle facial skeleton. His first toe was much shorter than his second, with a large gap between them. Fingers were also very short.

### Cognitive and neuropsychiatric assessment

Clinical interview revealed a hyperactive (moving constantly on his chair) and impulsive boy with poor attention. He attempted to communicate but was hindered by phonological and articulatory impairments; at times he had excessive and disorganized speech, anxiety and emotional liability. He drew crude and violent fantasy scenes, saying that this calmed him down. He easily became frustrated and destroyed his drawings. His thinking was sometimes non-linear and disconnected with reality. He denied hallucinations when awake, but described difficulty falling asleep while seeing little animals trying to penetrate his head (possible hypnagogic phenomena).

Neurological examination was normal except for absent achilles and patellar reflexes. EEG while awake at 4 years-8 months revealed normal auditory and visual evoked potentials; slowing (predominantly on the right), with theta waves; and frontal monomorphic delta waves without paroxysmal activity. EEG at age 10 showed rare slow temporal theta waves, sometimes sharp. Prolonged waking and sleep EEG at 10 years-3 months revealed isolated sharp waves (while awake), but no continuous spike waves during sleep.

### Cognitive assessment

Table 1 The most striking feature was the discrepancy between verbal and non-verbal performance (Verbal Comprehension Index in the superior range, with borderline-impaired Working Memory and Processing Speed). Full-scale IQ computation was invalid because of the degree of heterogeneity. ASD symptoms were mild (subthreshold), with only the Developmental domain in the abnormal range. Notable abnormalities were seen for attention, capacity for inhibition, phonological skills, short- and long-term memory impairment, and motor skills.

**Table 1:** Cognitive and developmental assessments at age 10

Wechsler intelligence scale for children-IV	Scores	Comment
Verbal comprehension index	126 (96^th^ %ile)	Superior range
Similarities	16	(average range 8–12 on all WISC-IV subtests)
Vocabulary	10	
Comprehension	17	
Perceptual reasoning index	111 (77^th^ %ile)	High average range
Picture completion	10	
Block design	12	
Matrix reasoning	13	
Working memory index	70 (2^nd^ %ile)	Borderline range
Digit span	5	
Digit letter sequences	5	
Processing speed index	69 (2^nd^ %ile)	Impaired range
Coding	1	
Autism diagnostic interview - revised		
Social domain	8	Based on historical report for age 5. Only the Developmental domain is abnormal.
Communication domain and language	3	
Stereotyped behavior domain	3	
Developmental domain	5	
Test of everyday attention for children	<5%	
Selective visual attention	*<*4%	Notable impairment of attention and capacity for inhibition
Sustained attention	*<*2%	
Divided attention and intermodal processing	*<*2%	
Inhibition	*<*2%	
Attention control–cognitive flexibility	*<*3%	
Oral and written language, memory, attention		
Auditory verbal memory (L2MA)		
Word recall	−2.1 SD	Short- and long-term memory impairments
Word recall with visual cues	−5.6 SD	
Deferred word recall	−4.1 SD	
Sentence recall	−1.7 SD	
Phonology–naming/word repetition (N-EEL)		Performance at age 10 consistent with child at age 6
Monosyllabic A	−5 < *x* < −2.44 SD	
Monosyllabic B	−5 < *x* < −3.5 SD	
Plurisyllabic	−5 < *x* < − 5.1 SD	
Lexical reception and production (L2MA)		
Lex P	−1.3 SD	
Phonemic fluency	−2.8 SD	
Semantic fluency	−1.4 SD	
Antonyms	−1.2 SD	
Expression (ELO)		
Utterance production	−1 SD	
Sentence repetition	−5 SD	
Motor skills—NEPSY		Scaled scores; expected ≥8
Fingertip tapping	7	Heterogeneous profile with slow and imprecise motor coordination
Design copying	9	
Manual motor sequences	8	
Imitation of hand position	8	
Visual motor precision	3	
Finger discrimination	8	
Block construction	8	
Logical and mathematical abilities—UDN-II		
Developmental age 9–11, all subscales		Normal range
Psychomotor assessment		
Motor level and coordination (M-ABC)	−5.71 SD	
Hands movements imitation (EMG)	−5.29 SD	
Fingers movements imitation (EMG)	+0.64 SD	
Time and spatial organization (Bender)	DA = 6.5 years	
Geometry—REY-A FIGURE		
Visual- and spatial-integration	*<*2^nd^ percentile	
Visual- and spatial-memory skills	*<*2^nd^ percentile	

*WISC – IV* Wechsler intelligence scale for children – IV^27^

*ADI-R* autism diagnostic interview-revised^28^

*TEA-Ch* test of everyday attention for children^29^

*L2MA* spoken language, written language, memory, attention ^30^

*N-EEL* new tests for language assessment^31^

*ELO* oral language assessment^32^

*NEPSY* developmental NEuropsychological assessment^33^

*UDN-II* construction et utilisation du nombre-2^34^

*SD* standard deviation

*DA* developmental age

*M-ABC* movement assessment battery for children^35^

*EMG* evaluation de la motricité gnosopraxique distale^36^

*Bender* visual-motor test^37^

*Rey-A* Rey–Osterrieth complex figure test^38^

### Neuropsychiatric diagnoses

Final ICD-10 diagnoses included ADHD combined type, mixed specific developmental disorders including a prominent phonological deficit, and developmental coordination disorder with visual–motor impairments. The presentation resembled multiple complex developmental disorder^11,12^ (emotional regulation difficulties, impaired cognitive processing, confusion between reality and fantasy life, impaired social behavior and sensitivity) and multi-dimensional impairment.^13^

### Clinical genetic analysis

FISH probes showed no abnormalities (chromosomes 15q11, 17p11.2, 22q11 and 22q13). Fragile X syndrome was excluded by FMR1 PCR testing (exon 1). Comparative genomic hybridization (Agilent 2 × 105 array) detected an interstitial microduplication in distal 1q21.1 (144,940,840-146,290,831 bp, hg18), confirmed by FISH (BAC 533N14, Kreatech). Mother did not carry the duplication.

## Molecular analysis

**Fig. 1:**
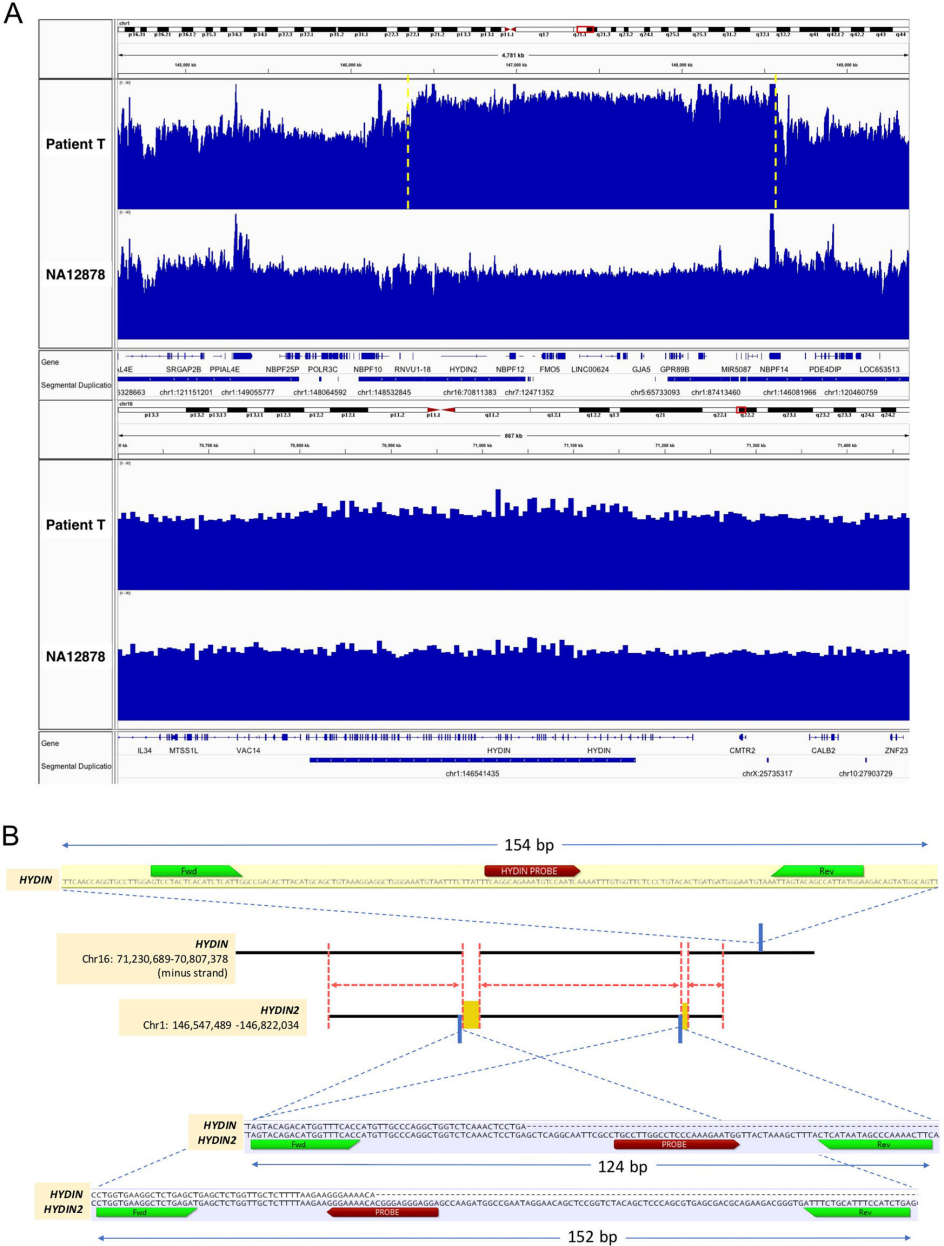
The patient’s 1q21.1 duplication CNV and the ddPCR assay strategy for *HYDIN2*. **a** Read depth of ~40× whole-genome sequencing across 1q21.1 (top panel) and 16q22.2 (bottom panel) for Patient T and HapMap DNA NA12878, plotted using Integrative Genomics Viewer.^24^ CNVnator^16^ called a heterozygous duplication CNV (copy number = 3, yellow dashed vertical lines) on chr1:146370001-148580000 (2.21 Mb, hg38) for Patient T and no CNV for NA12878. No CNV is observed over *HYDIN* on 16q22.2 (chr16:70700000-71400000) for either individual. *Y*-axis: read count from 0–80. **b** Shown is the region of overlap between the paralogous genes *HYDIN2* (1q21.1) and *HYDIN* (16q22.2, minus strand). Across the ~274 kb overlapping region, the two genes are estimated to share 99.4% sequence identity, with 264 Indels and 2049 mismatched bases.^9,25,26^ A detailed analysis of the evolution of *HYDIN2* demonstrated a more complex set of structural changes than can be shown here.^9^ Also shown are the three regions targeted by the ddPCR copy number assays reported here: the two insertions (1 and 6 kbp) that are unique to *HYDIN2*, and a segment of unique sequence in *HYDIN* (outside the overlap region)

Because this patient had preserved verbal ability and normal head circumference, we wanted to establish definitively whether *HYDIN2* was duplicated. *HYDIN2* is a human-specific gene created by an incomplete duplication of the *HYDIN* ancestral gene ~3.2 mya.^9,14^ *HYDIN2* lacks the first five and last two *HYDIN* exons.^9^ The sequence homology (Fig. 1) leads to incorrect CNV calls over *HYDIN* due to 1q21.1 CNVs,^5^ and the flanking SDs make it difficult to resolve CNV boundaries.

### Whole-genome sequencing and CNV analysis

We carried out 2 × 151 bp (350 bp insert) Illumina whole-genome sequencing (WGS) and obtained 42× genome coverage. After removal of PCR duplicates, CNV analysis based on sequencing read-depth^15^ was implemented on hg38 alignment data using the union of calls from CNVnator^16^ and ERDS.^17^ A 2.21 Mb duplication was detected in 1q21.1 (chr1: 146,370,001-148,580,000, hg38, by WGS (Fig. 1a). We found no other rare CNVs with known or likely neuropsychiatric consequences and also no loss-of-function mutations in *HYDIN2* or other apparent functional relevance to Patient T’s phenotype (see Supplementary Methods for details of sequencing and of CNV and SNV/indel calling and annotation). Comparison of *HYDIN2* vs. *HYDIN* sequence identified 1 kb and 6.1 kb “insertions” as the largest *HYDIN2*-specific segments. To estimate *HYDIN2* CN, we determined that 573 of T’s sequencing reads mapped to the 1 kb insertion vs. 369 in HapMap DNA NA12878 sequenced at 41×. The ratio of 185 suggested a heterozygous *HYDIN2* duplication.

### ddPCR copy number assays of *HYDIN* and *HYDIN2*

We developed ddPCR assays to measure these CNs more rigorously.^18,19^ We designed Taqman probes and corresponding forward and reverse primers targeting the 1 kb and 6.1 kb *HYDIN2* unique regions and a 154 bp segment of *HYDIN* outside the *HYDIN2* overlap region (Fig. 1b). The three ddPCR assays were successfully designed to quantify CN relative to reference gene *RPP30*, using a previously described algorithm.^20^

Performance of these assays was validated (Table S1 and S2) using 43 samples from the independent Molecular Genetics of Schizophrenia cohort^7^ with and without Affymetrix 6.0 microarray evidence for 1q21.1 CNVs (originally called with Birdsuite 2.0^21^). (The two *HYDIN2* ddPCR CN assays were always concordant.)

● Twelve subjects had microarray calls of heterozygous distal 1q21.1 CNVs (7 duplications and 4 deletions in individuals with schizophrenia; 1 deletion in a control), of which 10 also had CNV calls including *HYDIN* on 16q22.2. By ddPCR, all had *HYDIN* CN = 2. For *HYDIN2*, 10 had CN concordant with the microarray calls; 1 had CN = 2 which was consistent with the start position of the microarray-based duplication (chr1:144,943,150 bp, hg18), distal to the location of *HYDIN2*; and 1 deletion case had CN = 2 by ddPCR, but a sample swap was established by comparing genotypes from the microarray vs. from WGS.

● Seven (5 schizophrenia cases, 2 controls) had CNV calls in 16q22.2 over *HYDIN*, but without 1q21.1 CNVs. By ddPCR, all had *HYDIN* CN = 2, but 6 had *HYDIN2* duplications, suggesting the presence of 1q21.1 duplications (or other re-arrangements) too small to be reliably detected by microarrays. The exact nature of these variants is not yet determined.

● Twenty-four samples were selected with no microarray evidence of 1q21.1 or 16q.22.2 CNVs (the next two specimens on a plate after each 1q21.1 CNV sample). By ddPCR, all had CN = 2 for *HYDIN* and *HYDIN2*.

Ratios vs. *RPP30* were within tight ranges, permitting unambiguous CN calls: 45.4–49% for CN = 1 (*N* = 8); 89.4–102.6% for CN = 2 (*N* = 54); 140.3–152% for CN = 3 (*N* = 14).

### Heterozygous duplication of *HYDIN2* in Patient T

By ddPCR, the patient was found to have a heterozygous duplication of *HYDIN2* (CN = 3), while *HYDIN* CN was 2. For *HYDIN2* assays 1 and 2, ratios (vs. *RPP30*) were 1.48 (95% CI 1.43–1.53) and 1.52 (95% CI 1.47–1.57). For *HYDIN*, the ratio was 0.97 (95% CI 0.94–1.02).

## Discussion

This is the first report of a 1q21.1 duplication carrier with high verbal and low performance IQ. Previous reports noted that developmental and speech delays, intellectual disability and learning disabilities are common in 1q21.1 duplication cases,^2–8^ but verbal-performance discrepancies were not mentioned. It is unclear whether this feature is rare, or whether it escaped attention in previous reports which were not focused on comprehensive profiling of individual cases. This case also highlights the presence of motor coordination, articulatory deficits (verbal dyspraxia) and phonological disorders. Phonological deficits were specifically noted by Bernier et al.,^8^ and Nevado et al.^6^ noted an overall phenotypic similarity between 1q21.1 deletion/duplication and 22q11.2 deletion syndromes in which speech disorders are particularly common.^22^ Motor difficulties that affect gestural coordination and speech can have a global impact on functioning because of difficulties with socialization and emotional interaction.^23^ This case is an example of the great variability of the 1q21.1 phenotype, and it illustrates why comprehensive neuropsychological and behavioral evaluations are needed to guide individualized, multidisciplinary treatment programs.

Whole-genome sequencing did not reveal a molecular basis for the unusual phenotype seen here: the duplication had typical boundaries; no additional neuropsychiatric CNV was detected; and we did not observe notable functional rare variants. We studied *HYDIN2* further because it was hypothesized to contribute to cognitive and head size changes in 1q21.1 CNV syndromes.^5^ Our patient had normal head size and preserved verbal capacities, thus we wanted to determine definitively whether *HYDIN2* was duplicated. We developed two alternative ddPCR copy number assays of *HYDIN2* and one of *HYDIN*; both were concordant with results of dense microarray-based CNV analyses. These assays confirmed *HYDIN2* duplication in patient T.

While our ddPCR analyses were in progress, Dougherty et al. published an elegant analysis of the evolution of *HYDIN2*.^9^ To detect structural genomic changes, they evaluated CN using 153 molecular inversion probe (MIP) analyses of single-nucleotide differences between *HYDIN* and *HYDIN2* in 6055 individuals. *HYDIN2* was not part of 3 of 25 known 1q21.1 duplications and of 3 of 49 known deletions (consistent with the MGS dataset in which 1 of 7 duplications was distal to *HYDIN2*). They found that these 6 “atypical” cases had the expected head size changes (microcephaly with deletions, macrocephaly with duplications), suggesting that *HYDIN2* may not be involved in these changes. This MIP panel permits more detailed localization of CN change across *HYDIN2*, but in most cases, *HYDIN2* CN can be reliably evaluated with one or both of our ddPCR assays, or with a subset of the above-mentioned MIP assays. Further research will be needed to determine the phenotypic effects of copy number changes in *HYDIN2*.

## Electronic supplementary material

Supplementary Methods

Supplementary Tables

## Data Availability

The MGS sample was consented for deposition of anonymized clinical information and biomaterials by the National Institute of Mental Health. DNA, clinical information and existing GWAS and CNV data for this sample are available to qualified scientists from the U.S. National Center for Biotechnology Information-dbGAP repository for two data subsets which together, comprise the entire MGS cohort: GAIN (https://www.ncbi.nlm.nih.gov/projects/gap/cgi-bin/study.cgi?study_id = phs000021.v2.p1) and NON-GAIN (https://www.ncbi.nlm.nih.gov/projects/gap/cgi-bin/study.cgi?study_id = phs000167.v1.p1). Genome-wide transcriptome data are also available for a subset https://www.ncbi.nlm.nih.gov/projects/gap/cgi-bin/study.cgi?study_id = phs000775.v1.p1. Further information is available from the NIMH center for collaborative genomic studies on mental disorders (nimhgenetics.org). Please note that the informed consent from the guardian of Patient T did not include permission to deposit genetic data in a public repository. Investigators conducting related research may contact the present authors to discuss requests for additional information.

## Electronic supplementary material

**Supplementary information** accompanies the paper on the *npj Genomic Medicine* website (10.1038/s41525-018-0059-2).
