# Supplementary material for: 1q21.1 microduplication: large verbal–nonverbal performance discrepancy and ddPCR assays of HYDIN/HYDIN2 copy number
Source: NPJ Genom Med. 2018 Aug 22;3:24. doi: 10.1038/s41525-018-0059-2 (PMC6105585; doi:10.1038/s41525-018-0059-2)
Supplement: Supplementary file 1 — Supplementary Methods [file 41525_2018_59_MOESM1_ESM.pdf]

Supplementary Methods

Illumina whole-genome sequencing, QC, alignment, coverage, and visualization. Patient T genomic DNA was sent to Macrogen (Rockville, MD, USA) for standard Illumina (San Diego, CA) short-insert (350 bp) whole-genome sequencing library preparation and sequencing. One sequencing library was prepared and sequenced (2X151bp) on one lane of the Illumina HiSeq X Ten to achieve >30X coverage. NA12878 350 bp short-insert (2X151 bp) sequencing data generated from HiSeq X was obtained from Illumina Public Data (https://basespace.illumina.com/datacentral) (Run ID: Validationrun5_Nano350_MN). Reads from Lane #8 (>30X coverage) from this run was used for analysis. Short-insert (350 bp) whole-genome sequencing library was prepared from 100 ng of DL070-D05-03C22085 DNA using the Kapa Hyper Prep kit from Kapa Biosystems (Wilmington, MA, USA), PCR amplified for 5 cycles and sequenced on the NextSeq 500 from Illumina (San Diego, CA, US) using the NextSeq Mid-Output Kit (FC-404-2003).

We used FastQC (http://www.bioinformatics.babraham.ac.uk/projects/fastqc) to perform QC on the raw sequencing data obtained for Patient T. Overall, the median sequencing base quality score remained >q30 across all 2x151 cycles. The percent base content falls within the expected GC-percentages for a human genome, and there were no overrepresented sequences (no adapter contamination). The sequence duplication levels are ~10% as expected. No sequence trimming based on adapter contamination or low read quality was necessary. We then proceeded with sequencing alignment.

All reads were aligned to the human reference genome (hg38) using BWA-MEM [1] followed by marking and removal of duplicates using Picard tools (version 1.52) (http://broadinstitute.github.io/picard/) and Samtools (version 1.2) [2] respectively. Sequencing alignment coverage was calculated using the *coverage* function from BEDTools version 2.26 [3]. Read coverage of all sequencing alignments were visualized Integrative Genomics Viewer (version 2.3.82) [4].

Sequencing-based CNV analysis. Read-depth analysis [5] was implemented on the whole-genome sequencing alignment BAM file of Patient T to identify CNVs. CNVnator (version 0.2.7) [6] was used to first extract reads followed by histogram generation, statistics calculation and signal partitioning (using a bin size of 5 kbp). Final CNV calling using CNVnator was run using the "-unique" option. Another read-depth analysis tool, ERDS [7], was also used to call CNVs in Patient T with default parameters, and only "*PRECISE*" calls (as indicated by the *INFO* column in the output) were retained as the final calls. We performed further filtering on the ***union*** of the CNVnator and ERDS callsets. The rationale for this was that, to ensure that we did not miss a CNV that might have contributed to the patient's phenotype, the goal should be maximum sensitivity rather than specificity. If any possibly contributory CNV was detected, we could then have considered further validation steps.

The union callset was compliled by computing the percent overlap between pairs of CNVs with any overlap. If overlap was 50% in both directions (i.e., the ERDS call contained >50% of the CNVnator call, ***and*** the CNVnator call contained >50% of the ERDS call), the two calls were considered the same CNV, and the ERDS boundaries were retained because the ERDS algorithm is generally considered to be more accurate in determining boundaries. If the overlap in one or both directions was <50%, ***both*** CNVs were retained, again in the interests of maximizing sensitivity (this actually occurred only for a single pair of calls). CNVs made by only one caller were also retained.

CNVs were excluded if >50% of their length overlapped with reference gaps (hg38; downloaded from UCSC Genome Browser [8,9] and the ENCODE blacklist regions [10]). These blacklist regions are prone to produce artificially high coverage due to excessive unstructured

anomalous alignment. Note that CNV calls in telomeric and centromeric regions were also excluded by this procedure and by the other criteria described below).

This procedure resulted in 266 post-QC rare CNV calls, including 60 made by both callers with >50% reciprocal overlap as described above, 149 only by CNVnator, and 57 only by ERDS. There was only 1 pair of retained overlapping calls (because overlap was <50% in each direction).

We then excluded CNVs according to the following criteria:

1. Common CNVs (>1% in frequency) in any of the 19 Database of Genomic Variants (DGV[15]) cohorts with sample size >265 (April, 2018). (Note that these are control rather than disease cohorts.) Given their frequency, these CNVs are likely to be neutral or nearly neutral polymorphisms in the population and unlikely to be high-penetrance risk factors for disease or severe impairment. (165 deletions and 34 duplications excluded.)
2. CNVs that contain common CNVs regions (as defined in point 1 above). These CNVs could represent variations in the boundaries of the common CNV using the present WGS and calling methods. (8 deletions and 3 duplications excluded.)
3. CNVs with >50% of length overlapping segmental duplications (8 deletions, 14 duplications), pseudoautosomal regions (PAR1, PAR2; 2 deletions, 3 duplications) (because these regions are considered prone to false positives due to mapping issues), or chromosome 22p regions which are considered poorly annotated on the reference genome leading to unreliable mapping of reads (3 deletions, 1 duplication.

After the above filtering, 13 deletions and 11 duplications were retained as valid rare CNV calls. Of these, 20 were intergenic (some of them close to, but not overlapping, centromeres); only 4 (all deletions) overlapped with annotated genes (ASCL3, C14orf177, TPO, and ZNF718), none of which have known functional relevance for cognition, neuronal function or development. The only possibly relevant gene in the list is ASCL3: it is listed on Entrez Gene (accessed on genecards.org) as being possibly associated with Leukoencephalopathy With Vanishing White Matter, a very rare autosomal recessive, degenerative brain disease. The link on genecards.org is to http://www.malacards.org/card/leukoencephalopathy_with_vanishing_white_matter which in turn links to
https://diseases.jensenlab.org/Entity?order=textmining,knowledge,experiments&textmining=10&knowledge=10&experiments=10&type1=9606&type2=-26&id1=ENSP00000318846
and
https://diseases.jensenlab.org/Entity?documents=10&type1=9606&id1=ENSP00000318846&type2=-26&id2=DOID:0060868, from a website that lists bioinformatically-inferred (i.e., hypothetical) associations between genes and diseases, but no evidence is provided for this particular inference, and the confidence level is given as 2 out of 5 stars.

We therefore concluded that it was unlikely that Patient T's unusual neurodevelopmental phenotype was caused by any detected CNV other than the 1q21.1 duplication.

Analysis of possible loss of function mutations (SNVs & indels). The alignment BAM file of Patient T was first locally realigned around indels and had base quality scores recalibrated (*maximum_cycle_value=500, cov={ReadGroupCovariate, QualityScoreCovariate, CycleCovariate, ContextCovariate}*) using Genome Analysis Tool Kit (GATK version 3.7) [16,17]. The Haplotypecaller function from GATK was then used to identify SNV and indel variants using parameters *stand_emit_conf*=0.1, *variant_index_type*=LINEAR, *variant_index_parameter*=128000. The resulting variant scores were then recalibrated using the hg38 versions of training datasets (Mills and 1000 Genomes Gold Standard indels [18], dbSNP144 [19], HapMap 3.3 [20], Omni 2.5 genotypes, and 1000 Genomes Phase 1 [13]), as recommended by GATK Best Practices [17,21] and filtered at VQSR tranche = 90.0 in order to retain a low number of

false positive SNVs at the expense of a higher number of false negatives. Variants were then annotated with dbSNP144 [19].

To analyze for possible loss-of-function mutations, only exonic and splice-site variants were kept. Variants with frequency >1% in African, European or Asian HapMap [20] populations were also removed. Next, only variants (as annotated by SeattleSeq [22]) that cause missense, frameshift, splice-site change, and stop codon loss/gain were retained, resulting in 159 unique rare, potentially functional variants that were retained. Only six of these are annotated for phenotypes in relevant databases. Five of these variants are annotated in the Human Gene Mutation Database-Professional version (accessed via the Lane Library at Stanford University)[23], and one in ClinVar[24]. None of these annotated variants have any known relevance for the clinical symptoms manifested in Patient T.

Droplet digital PCR experimental design. Gene sequences of *HYDIN* and *HYDIN2* from hg38 reference assembly were obtained from UCSC Genome Browser [8]. Pairwise alignment of *HYDIN* and *HYDIN2* gene sequences were performed using Geneious software version 10 [25] (**Figure 1B**). All *HYDIN2* sequences are duplicated in *HYDIN* with the exception of two unique 1 kb and 6 kb "insertions" present within *HYDIN2* but not *HYDIN* (**Figure 1B**). To design Taqman probes targeting *HYDIN2*, 200 bp upstream and downstream of the 5' end junction of each of these unique "insertions" was extracted to serve as template sequence, and to design a Taqman probe targeting *HYDIN*, a 1 kb stretch of sequence towards the 3' end of *HYDIN* that is at least 5 kb away from sequences duplicated in *HYDIN2* was selected to use as the template . *HYDIN* Taqman probes and corresponding PCR primers were designed using Primer3 [26] with the following design parameters for probes: 18 to 22 bp in length, 60 to 65 °C for T_m, 0 to −9 kcal/mol for dimer ΔG, no G at the 5' end of the sequence, and stretches of more than 4 Cs or Gs were avoided. The reverse complement of the probe sequence was used if more Cs than Gs were present in the reverse complement sequence. PCR primers were designed to amplify 100 – 200 bp amplicons with T_m 5 to 10 °C lower than the corresponding Taqman probe. Primers and probes were ordered from IDT (Redwood City, CA, USA) with 5' FAM fluorophore and 3' ZEN Quencher modifications added to the probes. The sequences of primers and probes are listed in the table below. Synthesized double-stranded DNA containing the expected amplicon sequences for all the ddPCR assays [27] were ordered from IDT to use as positive controls and to determine the optimal annealing ddPCR annealing temperature for each assay. The optimal annealing temperatures were determined as 55 °C for the *HYDIN2* assays and 52 °C for the *HYDIN* assay. Droplets were generated using the QX200 Droplet Generator and QX200 Droplet Reader respectively from Bio-Rad (Hercules, CA, USA) following standard manufacturer's protocol [27]. A total of 40 cycles were performed for all ddPCR assays, and 20 ng of genomic DNA was used with all primers and probe concentrations at 250 nM. *RPP30* primers and probes [27] were used as reference for all assays. Quantification of target copy number was performed using a previously described algorithm [28].

HYDIN2 Assay 1	Forward primer	AGTACAGACATGGTTTCACC
	Reverse primer	GAAGTTTTGGGCTATTATGAG
	Probe	TGCCTTGGCCTCCCAAAGAATGG
HYDIN2 Assay 2	Forward primer	CTGGTGAAGGCTCTGAGAT
	Reverse primer	TCAGATGGAAATGCAGAAAT
	Probe	TCCTCCCTCCCGTGTTTTCCC
HYDIN Assay	Forward primer	GTCCTACTCACATCTCATTG
	Reverse primer	TCCATAATGGCTGTACTAAT
	Probe	TCAGGCAGAAATGTCCAATCA

SNP analysis of "DL070-D05-03C22085" to confirm sample swap. "DL070-D05-03C22085" hg38 alignment BAM file was first locally realigned around indels and had base quality scores recalibrated (*maximum_cycle_value=500, cov={ReadGroupCovariate, QualityScoreCovariate, CycleCovariate, ContextCovariate}*) using Genome Analysis Tool Kit (GATK version 3.7) [16,17]. The Haplotypecaller function from GATK was then used to identify SNP variants using parameters *stand_emit_conf*=0.1, *variant_index_type*=LINEAR, *variant_index_parameter*=128000. The resulting variant scores were then recalibrated using the hg38 versions of training datasets (dbSNP 138 [19], HapMap 3.3 [20], Omni 2.5 genotypes, 1000 Genomes Phase 1 [13]), as recommended by GATK Best Practices [17,21] and filtered at VQSR tranche = 90.0 in order to retain a low number of false positive SNPs at the expense of a higher number of false negatives. SNPs were annotated with dbSNP138 [19]. The resulting SNPs from GATK Haplotypecaller analysis were then converted from VCF format to PLINK [29] file formats using vcftools (version 0.1.13) [30]. The hg38 coordinates were then converted to hg18 using the LiftOver tool from UCSC Genome Browser [31].

SUPPLEMENTARY REFERENCES

1. Li, H. & Durbin, R. Fast and accurate short read alignment with Burrows-Wheeler transform. *Bioinformatics* **25,** 1754–60 (2009).
2. Li, H. *et al.* The Sequence Alignment/Map format and SAMtools. *Bioinformatics* **25,** 2078–9 (2009).
3. Quinlan, A. R. & Hall, I. M. BEDTools: A flexible suite of utilities for comparing genomic features. *Bioinformatics* **26,** 841–842 (2010).
4. Robinson, J. T. *et al.* Integrative genomics viewer. *Nat. Biotechnol.* **29,** 24–26 (2011).
5. Alkan, C., Coe, B. P. & Eichler, E. E. Genome structural variation discovery and genotyping. *Nat. Rev. Genet.* **12,** 363–376 (2011).
6. Abyzov, A., Urban, A. E., Snyder, M. & Gerstein, M. CNVnator: An approach to discover, genotype, and characterize typical and atypical CNVs from family and population genome sequencing. *Genome Res.* **21,** 974–984 (2011).
7. Zhu, M. *et al.* Using ERDS to infer copy-number variants in high-coverage genomes. *Am. J. Hum. Genet.* **91,** 408–21 (2012).
8. Kent, W. J. *et al.* The human genome browser at UCSC. *Genome Res.* **12,** 996–1006 (2002).
9. Karolchik, D. *et al.* The UCSC Table Browser data retrieval tool. *Nucleic Acids Res.* **32,** D493-6 (2004).
10. ENCODE Project Consortium. An integrated encyclopedia of DNA elements in the human genome. *Nature* **489,** 57–74 (2012).
11. Cooper, G. M. *et al.* A copy number variation morbidity map of developmental delay. *Nat. Genet.* **43,** 838–46 (2011).
12. Mills, R. E. *et al.* Mapping copy number variation by population-scale genome sequencing. *Nature* **470,** 59–65 (2011).
13. 1000 Genomes Project Consortium *et al.* An integrated map of genetic variation from 1,092 human genomes. *Nature* **491,** 56–65 (2012).
14. Coe, B. P. *et al.* Refining analyses of copy number variation identifies specific genes associated with developmental delay. *Nat. Genet.* **46,** 1063–71 (2014).
15. MacDonald, J. R., Ziman, R., Yuen, R. K. C., Feuk, L. & Scherer, S. W. The Database of Genomic Variants: a curated collection of structural variation in the human genome. *Nucleic Acids Res.* **42,** D986-92 (2014).
16. McKenna, A. *et al.* The Genome Analysis Toolkit: a MapReduce framework for analyzing next-generation DNA sequencing data. *Genome Res.* **20,** 1297–303 (2010).

17. DePristo, M. a *et al.* A framework for variation discovery and genotyping using next-generation DNA sequencing data. *Nat Genet* **43,** 491–8 (2011).
18. Mills, R. E. *et al.* Natural genetic variation caused by small insertions and deletions in the human genome. *Genome Res.* **21,** 830–9 (2011).
19. Sherry, S. T. *et al.* dbSNP: the NCBI database of genetic variation. *Nucleic Acids Res.* **29,** 308–11 (2001).
20. International HapMap Consortium. A haplotype map of the human genome. *Nature* **437,** 1299–320 (2005).
21. Van der Auwera, G. A. *et al.* From FastQ data to high confidence variant calls: the Genome Analysis Toolkit best practices pipeline. *Curr. Protoc. Bioinforma.* **43,** 11.10.1-33 (2013).
22. Ng, S. B. *et al.* Targeted capture and massively parallel sequencing of 12 human exomes. *Nature* **461,** 272–6 (2009).
23. Stenson, P. D. *et al.* The Human Gene Mutation Database: building a comprehensive mutation repository for clinical and molecular genetics, diagnostic testing and personalized genomic medicine. *Hum. Genet.* **133,** 1–9 (2014).
24. Landrum, M. J. *et al.* ClinVar: public archive of relationships among sequence variation and human phenotype. *Nucleic Acids Res.* **42,** D980-5 (2014).
25. Kearse, M. *et al.* Geneious Basic: an integrated and extendable desktop software platform for the organization and analysis of sequence data. *Bioinformatics* **28,** 1647–9 (2012).
26. Untergasser, A. *et al.* Primer3—new capabilities and interfaces. *Nucleic Acids Res.* **40,** e115–e115 (2012).
27. Hindson, B. J. *et al.* High-throughput droplet digital PCR system for absolute quantitation of DNA copy number. *Anal. Chem.* **83,** 8604–10 (2011).
28. Dube, S., Qin, J. & Ramakrishnan, R. Mathematical analysis of copy number variation in a DNA sample using digital PCR on a nanofluidic device. *PLoS One* **3,** e2876 (2008).
29. Purcell, S. *et al.* PLINK: a tool set for whole-genome association and population-based linkage analyses. *Am. J. Hum. Genet.* **81,** 559–75 (2007).
30. Danecek, P. *et al.* The variant call format and VCFtools. *Bioinformatics* **27,** 2156–2158 (2011).
31. Kuhn, R. M., Haussler, D. & Kent, W. J. The UCSC genome browser and associated tools. *Brief. Bioinform.* **14,** 144–161 (2013).
